# Aspirin administration from early pregnancy versus initiation after 11 weeks of gestation for prevention of pre-eclampsia in high-risk pregnant women: Study protocol for randomized controlled trial

**DOI:** 10.18502/ijrm.v22i1.15244

**Published:** 2024-02-23

**Authors:** Sedigheh Hantoushzadeh, Arezoo Behzadian, Mohammad Mehdi Hasheminejad, Faezeh Hasheminejad, Alireza Helal Birjandi, Mojtaba Akbari, Marjan Ghaemi

**Affiliations:** ^1^Vali-E-Asr Reproductive Health Research Center, Family Health Research Institute, Imam Khomeini Hospital Complex, Tehran University of Medical Sciences, Tehran, Iran.; ^2^Department of Perinatology, Vali-E-Asr Hospital, Imam Khomeini Hospital Complex, Tehran, Iran.; ^3^Iran University of Medical Sciences, Tehran, Iran.; ^4^Asklepios Psychiatry Lower Saxony GmbH, Asklepios Specialist Clinic, Gottingen, Germany.; ^5^Hannover Medical School, Clinic for Urology, Hannover, Germany.; ^6^Isfahan Endocrine and Metabolism Research Center, Isfahan University of Medicine Sciences, Isfahan, Iran.; ^7^Vali-E-Asr Hospital, Imam Khomeini Hospital Complex, Iran.

**Keywords:** Pre-eclampsia, Aspirin, Color doppler ultrasonography, Pregnancy, First trimester, Pregnancy-associated plasma protein-A.

## Abstract

**Background:**

Pre-eclampsia (PE) is a multiorgan disorder that affects 2–5% of all pregnant women. Present recommendations for when to start aspirin in high-risk women are after 11 wk of gestation.

**Objective:**

We present a protocol to investigate the effectiveness of aspirin use from early pregnancy, which is a randomized controlled trial to assess whether prescribed low-dose aspirin from early pregnancy reduces the prevalence of early and late-onset PE. Additionally, to compare the effectiveness of aspirin administration before and after 11 wk in reducing the occurrence of PE?

**Materials and Methods:**

All pregnancies at risk of PE, according to demographic and midwifery history, who are referred to the Maternal-Fetal Clinic of Tehran University hospital, Tehran, Iran were invited to take part in the trial. The outcomes of pregnancy and newborns will be gathered and analyzed. The first registration for the pilot study was in January 2023, and the participants were recognized as high-risk for PE. In addition, enrollment in the main study will begin as of October 2023.

## 1. Introduction

Pre-eclampsia (PE) is a multiorgan disorder that occurs in 2–5% of pregnancies (1). The cytotrophoblasts infiltrate into the decidual portion of spiral arteries, but they do not reach the myometrial portion. This results in the vessels remaining narrow (2).

A study has shown that there is a positive feedback loop between endothelial dysfunction and oxidative stress in the placenta in PE caused by sFlt-1. The study found that this positive feedback loop activates the apoptosis pathway in trophoblasts in preeclamptic placentas. Additionally, it was discovered that low-dose aspirin (LDA) can alleviate oxidative stress and endothelial dysfunction mediated by sFlt-1 and inhibit apoptosis of trophoblasts in PE (3).

PE is associated with turnover and increased platelet-derived thromboxane levels. These findings have led to randomized controlled trials (RCT) examining LDA therapy at a dose of 150 mg in pregnancies at risk of the disease (4, 5).

In the ASPRE trial the aspirin for evidence-based PE, participants were randomized to take aspirin or a placebo in 11–14 wk. The authors concluded that LDA significantly reduces the rate of preterm PE (6). An RCT examined the effects and safety of LDA (81 mg/day) starting at 6 wk and 13 ${\;}^{6/7}$ wk in nulliparous women, in low and middle-income countries. They reported significant reductions in perinatal mortality, fetal loss, early preterm labor (PTL), and the incidence of hypertensive disorders (7). Meta-analyses suggest that taking LDA starting at or before 16 wk of pregnancy significantly reduces the risk of severe PE and other placenta-related complications (8).

The mechanism of LDA remains vague, but it could include a recovery of the remodelling of spiral arteries, and increase the uterine artery blood flow related to the improved transformation of spiral arteries (5).

Another study found that aspirin's effect on sFLT1 production from cytotrophoblasts is replicated through cytochrome c oxidase I inhibition but not COX2 inhibition (9).

Panagodage and colleagues hypothesized that LDA improves trophoblast function affected by PE by altering cytokine production and secretion, including placental growth factor, resulting in an overall positive effect on trophoblast cell function in vitro (10).

The safety of using LDA during pregnancy has been extensively studied and no significant adverse maternal or fetal/neonatal effects have been reported (8, 11).

The results of a RCT on the safety of using LDA during pregnancy show different findings from previous observational studies on aspirin use during the first trimester. The previous studies suggested an increased risk of certain birth defects such as gastroschisis, cryptorchidism, oral clefts, neural tube defects, microphthalmia, and limb body wall defects. However, the RCT findings are similar to other large studies that reported no association between aspirin use and birth defects overall. Additionally, no links between LDA and malformations have been reported. This could be due to the fact that LDA is broken down in the mother's liver, resulting in low fetal exposure (12).

No original RCT has been conducted on the effectiveness of LDA administration from early pregnancy vs. after 11 wk for the prevention of PE.

### Primary objective

To determine the effectiveness of early use of LDA in high-risk pregnancies in reducing the incidence of early and late-onset PE.

### Secondary objectives

  To determine the effect of LDA prescription during early pregnancy on adverse perinatal outcomes:

  Incidence of placental abruption (clinically or on placental examination)

  Incidence of small for gestational age (SGA) ( < 5 ${\;}^{\text{th}}$ centile) requiring delivery at < 34 wk and < 37 wk

  Incidence of stillbirth or intrauterine fetal death (IUFD) at ≥ 20 wk

  Incidence of spontaneous preterm delivery at < 34 wk and < 37 wk

  To determine the effect of LDA during early pregnancy on neonatal mortality and morbidity:

  Respiratory distress syndrome is described as the requirement for surfactant and ventilation due to prematurity

  Ventilation requirement is defined as the need for positive pressure

  Admission to neonatal intensive care unit (NICU)

## 2. Materials and Methods

### Trial design

The study has 2 portions: a pilot study with quality control of the study, and a parallel assessor-blind randomized trial (main RCT) by allocation ratio 1:1. According to the Gantt table, the study schedule is set out (Table I).

### Pilot study and quality control of the study

A pilot study is used before the start of the main RCT to assess the feasibility of recruitment and the ability of the center to conduct the RCT.

A total of 1686 pregnancies were screened and 200 high-risk women with PE were enrolled in the pilot study. The complications of the main RCT include the need to conduct periodical targeted monitoring during the study for quality control of the recruitment of participants, as well as the correct measurement of the uterine arteries pulsatility index (UtA-PI), biochemical screening tests, the appropriate follow-up of the participants, monitoring, the correct use of the medicine, and the evaluation of the quality of the data collection. Additionally, if necessary, we will implement strategies to improve study quality such as retraining (including health staff involved in the follow-up of the participants and UtA-PI measurement). Accordingly, the ability of the center and the study team to conduct this pilot trial has been evaluated and was successful. Recruitment for the pilot study was started in January 2023. The pilot study data will be analyzed and published.

**Table 1:** Gantt table (study schedule chart)


Time point**		**Enrolment**	**Allocation**			**Close-out**
		**10.01.2023**	**01.02.2023**	**01.03.2023**	**etc.**	**30.12.2023**
**Enrolment**				
**Eligibility screen**				
**Informed consent **				
**Allocation**				
**Interventions**					
**Assessments**				
	**List baseline variables**				
	**List outcome variables**				

### Participants

The participants are pregnant women who are at risk of PE based on their demographic characteristics and obstetrics history, according to the ACOG, USPST, and SMFM guidelines. During a structured clinical interview, all enrolled participants will be evaluated using a questionnaire designed by the perinatology fellowship senior based on the guidelines mentioned. All participants were involved in the research setting. Additionally, patients and their families were central to disseminating the baseline information to motivate community involvement during and beyond the study.

#### Inclusion criteria

I. No serious maternal illness or learning difficulties

II. Pregnancies at risk of PE conforming to the USPSTF (13), ACOG, and SMFM (14) guideline criteria:

If there are one or more of the following criteria (major criteria), participants are considered at high risk:

1) Prior pregnancy with PE particularly early-onset PE or with an adverse outcome

2) Chronic hypertension

3) Type 1 or 2 diabetes mellitus

4) Renal disease

5) Autoimmune disease (antiphospholipid syndrome, systemic lupus erythematosus)

6) Multifetal gestation

If there are 2 or more of the following criteria (moderate criteria), they are considered high-risk:

1) Nulliparity

2) Obesity (body mass index > 30 kg/m^2^)

3) Age ≥ 35 yr

4) Family history of PE in mother or sister

5) Obstetric risk factors (such as a history of low birth weight or SGA, and other adverse pregnancy outcomes, > 10 yr pregnancy interval)

6) Low socioeconomic level

7) In vitro fertilization (IVF)

#### Exclusion criteria

1) Hypersensitivity to nonsteroidal anti-inflammatory drug products

2) Asthma, rhinitis, and nasal polyps

3) Severe renal or severe hepatic impairment

4) History of gastrointestinal bleeding or active peptic ulcer disease

5) Vaginal bleeding or significant hematoma on ultrasound

6) Gestational age > 16 wk

### Sample size

The sample size estimation of the main RCT was based on data from the ASPRE trial (6). The results of this trial showed that preterm PE occurred in 1.6% of the intervention group as compared with 4.3% in the placebo group. We calculated that the enrollment of 1792 participants in a 1:1 ratio of distribution (896 participants in each group) would provide the trial with a power of 90% (alpha level of 5%). If we are allowed a 10% loss to follow-up, the sample size will reach 986 participants in each group. A total of 1972 eligible participants will be randomly selected to participate in the trial arms. Furthermore, we proposed a sample size of 200 high-risk pregnancies in the pilot study and quality control. By screening 1686 pregnant women who attended the Maternal-Fetal hospital Clinic, we achieved this sample size. From January 2023, after obtaining written conscious consent, the trial began with 100 participants in each group at a 1:1 ratio, randomly.

### Randomization

The participants of control and intervention groups were assigned randomly at a ratio 1:1.

#### Sequence generation

The method of assigning participants to study groups was performed in the form of block randomization (with random blocks of 4 alleles and with a size of 8), which was performed by the specialized random allocation software.

#### Allocation mechanism

Due to the non-blindness of the trial, concealment was not performed.

#### Implementation

The perinatology fellowship assistant randomly did the allocation. The registration of participants was done by the personnel of the Prenatal Clinic of Vali-E-Asr hospital.

### Blinding

Due to the nature of the intervention, neither participants nor researchers could be blinded to allocation. However, assessments will be conducted by a blind analyzer for trial allocation.

### Intervention

After the participant screening, written informed consent was obtained from them, which was approved by the Tehran University Ethics Committee, Tehran, Iran.

During the first visit, vital signs, including blood pressure, weight, and height for accounting for body mass index, were measured. A complete record of medication usage is obtained, particularly those that interact with aspirin, such as anticoagulants, antiplatelet agents such as clopidogrel, antidepressants, and corticosteroids, smoking, and alcohol consumption. 30 days after aspirin was administered, a follow-up telephone interview was conducted for side effects and adverse events. At each clinical visit, PE symptoms and the presence of edema were noted and recorded. In addition, participants were asked about the possible side effects of aspirin.

In the pilot study, 200 eligible participants were randomly selected (100 participants in each group). Individuals who received LDA before 11 wk of gestation (optimally from the detection of the fetal heartbeat by ultrasound exam) were considered the intervention group and those who receive LDA after 11 wk are the control group. In the control group, the cut-off gestational age to prescribe aspirin was 16 wk. In both groups, the initial dose of aspirin is 80 mg per day, and it will be continued until 36 wk or in the case of premature delivery.

The mean UtA-PI is measured simultaneously with nuchal translucency (NT) determined through a transabdominal ultrasound exam at 11–13 ${\;}^{6/7}$ wk. In addition, a combined aneuploidy screening test was requested by measuring the maternal serum-free beta-human chorionic gonadotropin (β-hCG) and pregnancy-associated plasma protein-A (PAPP-A) using the Cobas-E-411 analyzer.

If there are any criteria, the initial dose of aspirin (80 mg) is changed to 150 mg per day:

• PAPP-A < 0.4 in the first trimester (15)

• PE risk greater than or equal to 1/100 in the aneuploidy screening test (16)

• High resistance of uterine arteries in color Doppler ultrasound (the mean UTA-PI > 95 ${\;}^{\text{th}}$ percentile or bilateral notching) (17)

At 18–22 wk, the mean UtA-PI will be measured and recorded at the same time as the anomaly scan. Ultrasound examinations are performed by 2 trained prenatal fellowships that have Fetal Medicine Foundation certification.

During the trial, some participants may be excluded from the study due to reasons such as abortion or IUFD below 20 wk of gestation or major fetal abnormalities or side effects of aspirin or discontinue the intervention. We will use the CONSORT checklist when writing our report.

### Study assessment

**Figure 1:**
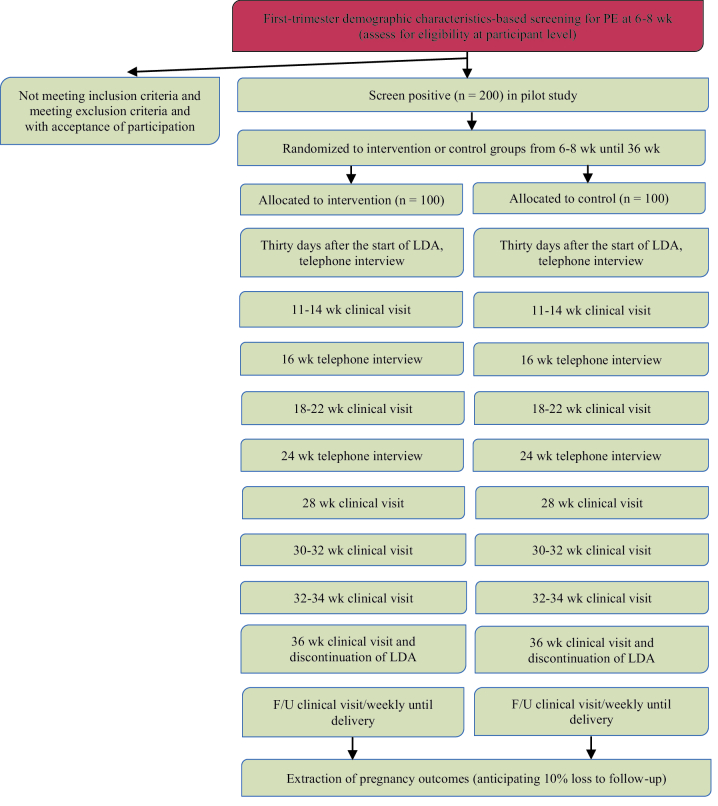
Flowchart of study assessment; the method of follow-up and evaluation of participants in both intervention and control groups is given in this chart. Wk: Weeks, F/U: Follow-up, LDA: Low-dose aspirin, PE: Pre-eclampsia.

The study procedure through visits is schemed in figure 1.

### Laboratory tests

At the same time, as the 11–14 wk ultrasound exam, a combined test will be requested to measure PAPP-A and beta-human chorionic gonadotropin using automated machines that provide reproducible results (the Cobas-E-411 analyzer, Ruche-Switzerland).

### Data collection

An electronic medical file was created for each patient to record demographic and obstetric information, physical examination results, laboratory tests, and imaging during each visit. In addition, all information related to unusual events such as obstetrical hospitalization, or aspirin side effects, and delivery information were recorded.

### Data collection methods

The participant's data will be entered into the information collection forms (Table II). Demographics, obstetrics characteristics, paraclinical results, delivery, neonate data, and perinatal outcomes will be entered into data collection forms. Finally, all the information will be entered in the electronic case report form which will be analyzed.

#### Consent for dissemination

Not enforceable- we have not rendered any identifying images or other personal, or clinical details of participants here.

#### Accessibility of data

This script constitutes the complete protocol. Following the completion of the study information used in this study will be obtained from the corresponding author upon rational request.

**Table 2:** Data collection form


**Patient information**	
**Maternal demographic characteristics**	
**1**	Record of hypertension in a prior pregnancy
**2**	Chronic hypertension
**3**	Autoimmune disease: APs, SLE
**4**	Renal disease
**5**	Type 1 or 2 diabetes mellitus
**6**	Multiple fetuses
**7**	Nulliparity
**8**	History of PE in the mother or sister
**9**	BMI ≥ 30
**10**	Age ≥ 35
**11**	Low level socioeconomic
**12**	IVF
**13**	Obstetrical risk factors included SGA, PTL
**Paraclinical data**	
**14**	PE risk in first-trimester aneuploidy screening test
**15**	PAPP-A
**16**	Mean UtA-PI of the first trimester
**17**	Mean UtA-PI of the second trimester
**Delivery, neonate data, and outcomes**	
**18**	Gestational diabetes under insulin
**19**	Gestational diabetes without insulin
**20**	Weight gain during the 1 ${\;}^{\text{st}}$ trimester
**21**	Weight gain during the 2 ${\;}^{\text{nd}}$ trimester
**22**	Weight gain during the 3 ${\;}^{\text{rd}}$ trimester
**23**	Total weight gain
**24**	Gestational age at delivery
**25**	Birth weight
**26**	Postpartum hemorrhage
**27**	Drug-related side effects and adverse events
**28**	Placenta abruption
**29**	Early-onset PE
**30**	Late-onset PE
**31**	The requirement for NICU admission or mechanical ventilation
**32**	Neonatal respiratory distress syndrome
**33**	Hospitalization for maternal and fetal observation
**34**	Spontaneous PTL < 34 wk
**35**	Spontaneous PTL < 37 wk
**36**	IUFD or stillbirth > 20 wk
APs: Anti-phospholipid syndrome, SLE: Systemic lupus erythematosus, PE: Pre-eclampsia, BMI: Body mass index, IVF: In vitro fertilization, SGA: Small for gestational age, PTL: Preterm labor, PAPP-A: Pregnancy-associated plasma-A, UtA-PI: Uterine artery pulsetillity index, NICU: Neonatal intensive care unit, IUFD: Intrauterine fetal demise	

### Outcomes

#### Primary outcome

The primary outcome is determining the occurrence of early-onset ( < 34 wk) and late-onset ( ≥ 34 wk) PE. PE is described by the International Society for the study of hypertension in pregnancy (18); as systolic blood pressure ≥ 140 mmHg and/or diastolic blood pressure ≥ 90 mmHg on at least 2 times 4 hr apart developing after 20 wk of gestation in previously normotensive women, and there should be proteinuria ≥ 300 mg in 24 hr, or urinary protein creatinine ratio ≥ 30 mg/mmol midstream, or catheter urine specimens if no 24-hr collection is available, or 2 readings of at least ++ on dipstick analysis of midstream, or in the absence of proteinuria, new-onset hypertension with any of the following:

- Thrombocytopenia (platelet count < 100000/µL)

- Renal insufficiency (serum creatinine > 1.1 mg/dL)

- Impaired liver function as described by liver transaminase levels at least twice the normal concentration

- Pulmonary edema

- Persistent cerebral or visual symptoms

PE symptoms are measured clinically in every visit and paraclinical tests are requested if symptoms are present.

During each visit, clinically determined PE symptoms shall be measured and paraclinical tests must be performed where symptomatic signs are present.

#### Secondary outcomes

1. Placental abruption (clinically or on placental examination), which is determined at the time of delivery.

2. SGA ( < 5 ${\;}^{\text{th}}$ centile) requiring delivery at < 34 wk and < 37 wk, which is measured by ultrasound.

3. Stillbirth or IUFD at ≥ 20 wk, which is determined by ultrasound.

4. Spontaneous preterm delivery at < 34 wk and < 37 wk, which is determined by clinical examination and ultrasound.

5. Respiratory distress syndrome, described as the requirement for surfactant and ventilation due to prematurity, which is diagnosed by examination at the time of birth.

6. Ventilation requirement is defined as the need for positive pressure.

7. Neonatal intensive care unit admission, which is diagnosed by examination at the time of birth.

8. Neonatal anomaly, especially gastroschisis.

### Gathering of outcomes

#### Gathering of pregnancy and neonatal outcomes

Data related to delivery and newborns will be collected through the patients and the hospital maternity records. The outcome of the participants will be assessed in terms of the incidence of early and late-onset PE, placental abruption, stillbirth, spontaneous premature delivery, postpartum hemorrhage, birth weight, infant respiratory distress at birth, need for mechanical ventilation, and admission to the NICU. If neonates are admitted to the NICU, neonatal outcomes will be collected from the discharge summary of the NICU.

#### Gathering of side effects and adverse incidents

Side effects are defined as any unwanted drug complications including hypersensitivity reactions, bleeding complications, headache, dizziness, nausea, vomiting, etc. Adverse events are defined as hospitalization for maternal and fetal observation, including bleeding course, spontaneous PTL, premature rupture of membranes, abortion, stillbirth, and the termination of pregnancy for fetal or maternal indications. Thirty days after the start of aspirin administration, a follow-up telephone interview was conducted with the participants to assess side effects and adverse events. If these events are considered a part of the normal development of the corresponding pregnancy, they will not be included in the category of drug-related complications but will be recorded in the clinical electronic records of the participants. If it is necessary to stop the drug permanently, the data will be placed in the data proportion of the discontinuation and will be removed from the final analysis.

### Monitoring

During the trial, some participants may be excluded from the study due to reasons such as abortion or IUFD below 20 wk of gestation or major fetal abnormalities or side effects of aspirin. In case of severe side effects and the need to discontinue the drug, the data will be placed in the data proportion of the discontinuation and will be removed from the final analysis. Since the trial is not blind for the researchers, they can access the results at any stage, due to the nature of the intervention.

### Safety considerations

A physician assesses the possible role of the LDA if there are any significant adverse effects within therapy. If it is approved, the patient will be omitted from the study. Despite recording all the lost data about outcomes and follow-ups, it will not be included in the analysis. The discontinuation of treatment for pregnancies will be expressed by reason and study group.

### Ethical considerations

The trial was verified by the ethics committee of the Tehran University of Medical Sciences, Tehran, Iran Institutional Review board (Code: IR.TUMS.IKHC.REC.1401.215) on October 18, 2022, and steered conforming to the Declaration of Helsinki and later reconsiderations.

Written informed consent was acquired from all participants by the interviewer who included perinatology fellowship or prenatal clinic treatment staff. Participants are informed that their participation is voluntary and they may withdraw from the trial at any time without negative consequences on their treatment. The trial was registered on January 7, 2023, at the Iranian Registry of Clinical Trial (http://www.irctir/; IRCT ID: IRCT20221126056611N1). The last registry update was confirmed on 2023–12-21.

### Trial status

Employment for the pilot study began in January 2023 and is expected to end by December 2023. Additionally, recruitment for the main RCT was started in October 2023.

### Statistical analysis

For all primary analyses, the intervention group will be compared to the control group. Statistical analyses will be performed using STATA statistical software version 17.0 (STATA Corp, Texas, USA) and SPSS version 25 (SPSS, Inc., Chicago, IL, USA). Descriptive data will be reported as the mean ± SD, median (IQR), or count (percent) as appropriate. The Shapiro-Wilk normality test will be applied to test the normality of the quantitative data. Continuous data will be compared between 2 groups using an independent sample *t* test or Mann-Whitney U test, and categorical data will be compared using the Chi-square test. Finally, to find the independent effect of aspirin treatment in high-risk participants a multiple logistic regression test will be used, in which the effect of aspirin treatment will be adjusted for the background variables of the participants that will differ between the 2 groups. The level of significance was < 0.05. A complete set of descriptive statistics will be produced for all variables overall and by the treatment group. Graphical displays will be produced as appropriate.

#### Secondary analysis

By using appropriate tests, the secondary outcomes will be compared between study groups. The p-value and 99% CI will be generated for the treatment results.

## 3. Discussion

Trophoblastic invasion into the myometrium portion of the spiral arterioles during normal placentation is performed in a 2-stage process. It starts at 6–8 wk of gestation and culminates at 16–20 wk. During this process, the spiral arteries are replaced with wide nonmuscular channels insensitive to vasomotor control. Incomplete deep placentation has been associated with a spectrum of pregnancy complications including PE (19).

PE is caused by the inability of trophoblast cells to invade the spiral arteries of the myometrial layer. Lyall and colleagues found that vascular spasms and inefficient blood circulation inside the fetoplacental unit are caused by ineffective trophoblast invasion (20).

The incidence of disease may be halved by LDA consumption before 16 wk of gestation (21). In one systematic review study, aspirin reduces the risk of preterm PE only when initiated at 16 wk of gestation and taken at a daily dose of 100 mg, but not term PE (22). Meta-analysis recommended that aspirin use before 17 wk decreases the risk of PE in SGA neonates (23). The mechanism of aspirin remains obscure, but it could include recovery of the alteration of uterine spiral arteries (22, 23). The level of platelet activation in pregnant women with PE is significantly higher than that in pregnant women without PE. This is shown by a shortened activated partial thromboplastin time and a hypercoagulable state. Aspirin can help regulate the balance between thromboxane and prostacyclin, and can also inhibit platelet aggregation. This can prevent the formation of small blood clots, reduce damage to organs caused by blood clots, and help to prevent PE (4, 5, 24).

Other studies found LDA improves altered cytokine production and has positive effects on sFTL1 production (9, 10).

We designed an RCT to investigate whether the earlier administration of aspirin from the first stage of spiral arteries invasion in the process of the placentation could further reduce the incidence of PE. We also speculate that aspirin administration at the same time as the spiral arteries transformation may be more effective in the prevention of PE and other complications related to these vessel disorders. In addition, this trial was designed to compare the effectiveness of aspirin administration at this time versus administration after 11 wk to prevent early and late-onset PE.

### Strengths and limitations of the trial

1. This is the large single-center RCT to compare the effect of aspirin administration from early pregnancy versus after 11 wk in women at risk of PE.

2. Screening and start of prevention with aspirin from the early stage of pregnancy in high-risk women can provide an opportunity for more effective prevention of PE.

3. Follow-up of newborns is only confined to the primary postnatal period for the time of stay in the hospital.

##  Data availability

Following the completion of the study, information used will be obtained from the corresponding author upon rational request.

##  Author contributions

SH, AB, FH, AHB, and MG created essential contributions to the opinion and scheme of the work. AB, MMH, FH, and AHB have an essential contribution to data accumulation. MA will analyze and interpret the information. AB, MMH, FH, AHB, and MG made the main contributions to writing the script. The writers contribute to the refinement of the study and verify the ultimate manuscript.

##  Conflict of Interest

The authors declare that there is no conflict of interest.
